# The Impact of the Thermal Seasons on Adenoid Size, Its Mucus Coverage and Otitis Media with Effusion: A Cohort Study

**DOI:** 10.3390/jcm10235603

**Published:** 2021-11-28

**Authors:** Krystyna Masna, Aleksander Zwierz, Krzysztof Domagalski, Paweł Burduk

**Affiliations:** 1 Department of Otolaryngology, Phoniatrics and Audiology, Faculty of Health Sciences, Ludwik Rydygier Collegium Medicum, Nicolaus Copernicus University, 85-168 Bydgoszcz, Poland; aleksanderzwierz@gmail.com (A.Z.); pawelburduk@icloud.com (P.B.); 2 Department of Immunology, Faculty of Biological and Veterinary Sciences, Nicolaus Copernicus University, 87-100 Toruń, Poland; krydom@umk.pl

**Keywords:** adenoid hypertrophy, seasons, mucus on adenoid, OME, tympanometry

## Abstract

Background: The purpose of this study is to analyze seasonal differences in adenoid size and related mucus levels via endoscopy, as well as to estimate changes in middle ear effusion via tympanometry. Methods: In 205 children with adenoid hypertrophy, endoscopic choanal assessment, adenoid hypertrophy assessment using the Bolesławska scale, and mucus coverage assessment using the MASNA scale were performed in two different thermal seasons, summer and winter. The study was conducted in two sequences of examination, summer to winter and winter to summer, constituting two separate groups. Additionally, in order to measure changes in middle ear effusion, tympanometry was performed. Results: Overall, 99 (48.29%) girls and 106 (51.71%) boys, age 2–12 (4.46 ± 1.56) were included in the study. The first group, examined in summer (S/W group), included 100 (48.78%) children, while the group first examined in winter (W/S group) contained 105 (51.22%) children. No significant relationship was observed between the respective degrees of adenoid hypertrophy as measures by the Bolesławska scale between the S/W and W/S groups in winter (*p* = 0.817) and in summer (*p* = 0.432). The degrees of mucus coverage of the adenoids using the MASNA scale and tympanograms were also comparable in summer (*p* = 0.382 and *p* = 0.757, respectively) and in winter (*p* = 0.315 and *p* = 0.252, respectively) between the S/W and W/S groups. In the total sample, analyses of the degrees of adenoid hypertrophy using the Bolesławska three-step scale for seasonality showed that patients analysed in the summer do not differ significantly when compared to patients analysed in the winter (4.39%/57.56%/38.05% vs. 4.88%/54.63%/40.49%, respectively; *p* = 0.565). In contrast, the amount of mucus on the adenoids increased in winter on the MASNA scale (*p* = 0.000759). In addition, the results of tympanometry showed deterioration of middle ear function in the winter (*p* = 0.0000149). Conclusions: The obtained results indicate that the thermal seasons did not influence the size of the pharyngeal tonsils. The increase and change in mucus coverage of the adenoids and deterioration of middle ear tympanometry in winter may be the cause of seasonal clinical deterioration in children, rather than tonsillar hypertrophy. The MASNA scale was found to be useful for comparing endoscopy results.

## 1. Introduction

Adenoid hypertrophy and otitis media with effusion are one of the most common childhood disorders, and can cause various ailments of the upper respiratory tract. The prevalence of adenoid hypertrophy in preschool and primary school children is estimated to be 49.7% [1]. The adenoid tissue may cause otitis media with effusion due to supranormal size, disruption of nasopharyngeal ventilation, Eustachian tube obstruction, mucus accumulation, oedema, and upper respiratory tract infection [2]. Other ethological factors are craniofacial malformations, mechanical obstruction of the nasopharynx, allergies, and immunodeficiency [3]. OME is reportedly experienced by 30–40% of all children [3]. The consequent obstruction of the upper respiratory tract causes mouth breathing, snoring, recurrent sinusitis, asthma, and sleep apnea, potentially leading to serious health damage including impaired development in children [1,4]. Quick and accurate diagnosis of the etiology can help in the proper treatment of obstructions [5]. Four or more episodes of recurrent purulent sinusitis, nasal obstruction, or otitis media with effusion in children aged four or older may indicate adenoid hypertrophy. There is a prevailing opinion that in cases of adenoid hypertrophy conservative treatment using topical steroids should be continued for at least two to three months; however, if there is no significant improvement, especially regarding sleep disturbances, adenoidectomy should be considered [6,7]. Various diagnostic methods can be used to assess the size of the pharyngeal tonsils, including nasopharyngeal lateral radiographs, computed tomography, videofluoroscopy, ultrasound, and mirror examination; the gold standard is flexible endoscopic examination [8,9,10]. Furthermore, a relationship between the size of the pharyngeal tonsils and the thermal seasons has been suggested, which may be based on the frequency of infections or allergy [11]. To the best of our knowledge and based on review of the literature in the PubMed database, there are no works analyzing adenoid size using flexible endoscopy in different thermal seasons, which prompted us to conduct this research.

In this study, we aimed to analyze changes in the size of the pharyngeal tonsils and their mucus depending on the thermal seasons by using endoscopic assessment. Furthermore, we evaluated changes in middle ear effusion by using tympanometry measurement. To better compare the population in this study with studies performed in other countries (often in different geographic zones with only two dominant seasons, winter and summer, such as the Mediterranean zone), we divided the year into two main seasons, winter and summer, considering the cutoff point for temperature to be 10 °C [12,13].

## 2. Materials and Methods

### 2.1. Study Population

205 children who visited a medical outpatient clinic with symptoms suggestive of chronic adenoid hypertrophy between 2016 and 2021 were included in the study. This was the first ENT consultation for those children; thus they were not given the standard conservative treatment for adenoid hypertrophy of a long course of antibiotics and steroids. Children with obstructive sleep apnea and upper airway obstruction, recurrent adenoid infection where two courses of antibiotics had failed, and four recurrent purulent rhinorrheas occurring in the preceding 12 months were included. Children with craniofacial anomalies, such as cleft lip/cleft palate, genetic diseases (Down Syndrome), neurological diseases, cardiovascular diseases, nasal septal deviation, nasal polyp or inferior turbinate hypertrophy, tympanic membrane perforation, and active upper respiratory infection within two weeks of enrolling in the study, or those who had previously undergone adenoidectomy or tympanostomy tube placement, were excluded from the study. We also excluded children with confirmed allergic rhinitis.

Each child was examined at least twice in two different seasons. The interval between the sequential seasonal examinations was 6 to 8 months. The summer examination occurred in the early summer, summer, and late summer (from 22 April to 12 October), when the average temperature in our region was above 10 °C, and the winter examination occurred in the early spring, spring, autumn, early winter, and winter (from 13 October to 21 April), when the average temperature was below 10 °C [11]. This study was a crossover group study, where approximately half of the children were first examined in winter and then in summer, and the remaining half were first examined in summer and then in winter. Initial assessment of each patient after enrollment in the study included a history and physical examination, parental questionnaire, flexible fiberoptic rhinoscopy, and tympanogram. After the first visit, patients received 12 weeks of conservative treatment with Mometasone Furoate nasal drops as a standard pharmacological treatment for adenoid hypertrophy [14,15]. Ethical approval for this study was obtained by the ethics committee of Nicolaus Copernicus University (KB 559/2021).

### 2.2. Endoscopy

Endoscopic examinations were performed by a pediatric otorhinolaryngologist (A.Z.) with 15 years of experience using the Karl Storz Tele Pack endoscopic system. This endoscopic system is equipped with a flexible nasopharyngoscope with a 2.8-mm outer diameter and 300-mm length. The percentage of obturation (adenoid-to-choanae ratio in percentage) of the choanae and mucus coverage of the adenoids were compared based on videoendoscopy with the freeze-frame option. Obstructions were assessed with an accuracy of up to 5%. Otoscopic examination was performed; if needed, the external auditory canal was cleaned, and tympanometry was performed using a GSI 39 Auto Tymp^TM^ by Grason-Stadler (Eden Prairie, MN, USA).

The percentage of choanal obstruction was measured and compared with the pretreatment value from the patient’s medical history score. The adenoid size and mucus coverage recorded on the endoscopic system were compared with those in the previously recorded video by a second doctor (K.M). If there was a discrepancy in the assessment, the score was reassessed by a third ENT doctor (P.B).

Change in adenoid size was considered as the percentage difference in the amount of nasopharyngeal obstruction by the tonsil. For statistical analysis to assign the grade of adenoid hypertrophy we applied the classification described by Boleslavska: grade I, adenoid tissue filling less than one-third of the vertical portion of the choanae; grade II, adenoid tissue filling between one-third and two-thirds of the choanae; and grade III, adenoid tissue filling more than two-thirds of the choanae [16].

**Figure 1:**
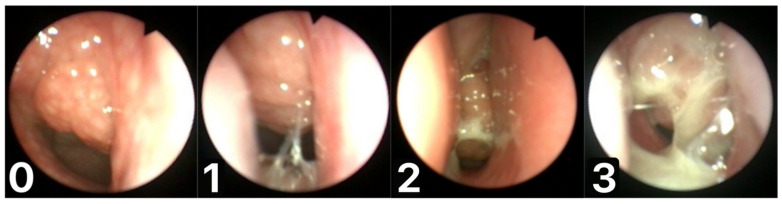
Mucus of Adenoid Scale by Nasopharyngoscopy Assessment (MASNA): (**0**)—no mucus; (**1**)—residue of clear watery mucus; (**2**)—some amount of dense mucus; (**3**)—copious thick dense mucus.

We devised a scale for assessing the mucus coverage of the adenoid, called the Mucus of Adenoid Scale by Nasopharyngoscopy Assessment (MASNA), which describes the amount of mucus covering the tonsil on a 4-point scale (0—no mucus, 1—residue of clear watery mucus, 2—some amount of dense mucus, 3—copious thick dense mucus) (Figure 1). The degree of seasonal change in adenoid mucus on the MASNA scale was compared for the total number of patients and between the designated groups. An increase in mucus coverage was represented by an increase in the degree of scale, while a decrease in the degree of scale implied reduced mucus coverage.

### 2.3. Tympanometry

The middle ear effusion in each ear was analyzed by tympanometry measurement and graphic tympanogram. The results were classified using the classification system for tympanograms developed by Liden and Jerger [17,18]. The sequence of saved tympanograms for each patient’s ear was first right, then left. We posited a type B tympanogram as the worst result, type C as bad, and A as good (normal). For further statistical analysis, we divided children into three groups, taking into consideration worse tympanogram in each child. Group A consisted of children with tympanogram A in both ears (AA), group C of children with tympanogram C (CC, AC, CA), and group B of children with tympanogram B (BB, BC, CB, AB, BA).

### 2.4. Ethics

All recorded videos of the nasopharynx were coded and blindly analyzed.

### 2.5. Statistical Analysis

We used descriptive statistics to present the characteristics of the study groups. We summarized continuous variables such as age and adenoid size through their means ± standard deviation (SD) and medians with 25th and 75th percentiles (Q25–Q75), and categorical variables such as gender, mucus coverage in the MASNA scale, adenoid size in the Boleslavska scale, and tympanograms using frequency counts and percentages. This study analyzed whether the seasonal order of the study, that is, winter to summer examination or summer to winter examination, had an impact on the results. In order to determine the effect of the sequence of examination on the clinical results, we compared qualitative data using the chi-squared method or Fisher’s exact test where appropriate, and quantitative data using Student’s *t*-test for independents variables. In order to assess the impact of the thermal seasons on adenoid size, adenoid mucilage coverage, and tympanograms, we evaluated the statistical significance by analysis for dependent variables. Continuous variables were compared with Student’s *t*-test test for paired samples. McNemar–Bowker tests were used for analysis of categorical variables.

For all these tests, two-tailed *p*-values were used and *p*-values < 0.05 were considered to be statistically significant. All statistical analyses were conducted with SPSS (Statistical Package for the Social Sciences version 26, Armonk, NY, USA) software.

## 3. Results

A total of 205 children with sequential seasonal ENT examinations were studied, 99 girls (48.29%) and 106 boys (51.71%), age 2–12 with a mean of4.46 ± 1.56 (Table 1). The study was conducted in two sequences of examination, summer to winter and vice versa, constituting two separate groups with comparable sample sizes. The group which was first examined in the summer (S/W group) included 100 children (48.78%), and group first examined in the winter (W/S group) included 105 children (51.22%).

For the next step of our analysis, we estimated associations between the sequence of examinations and demographical and clinical characteristics. These analyses were aimed at determining whether or not the obtained results depend on the season in which the patient’s clinical analysis was started. As shown in Table 2, there were no statistically significant differences in the demographical and clinical characteristics between the S/W and W/S groups. Both groups were similar in terms of age and gender. In both groups the mean adenoid size was similar, 61.70% and 64.05%, respectively, in winter was and 60.70% and 63.57%, respectively, in summer, and did not differ statistically significantly (*p* = 0.303 and *p* = 0.432, respectively, for winter and summer). The groups were also similar in terms of mucus coverage of adenoids and tympanograms. The degree of mucus coverage of adenoids using the MASNA scale was comparable. Comparing winter examinations between the group first examined in summer and the group first examined in winter, we did not find statistically significant differences (*p* = 0.315). Comparing summer examinations, the group first examined in summer and the group first examined in winter were again similar, and we found no statistically significant differences between the groups (*p* = 0.382). Moreover, the type and frequency of occurrence of different types of tympanograms were similar. There were no statistical significance between the S/W and W/S groups in tympanometry performed in winter (*p* = 0.254) and in summer (*p* = 0.757) (Table 2).

The primary analysis in this study focused on the association between thermal season and adenoid size, adenoid mucus coverage and tympanograms. We observed that in the summer first-degree of adenoid hypertrophy by the Bolesławska scale was present in 9 (4.39%) children, second-degree in 118 (57.56%) children, and third-degree in 78 (38.05%) children, whereas in winter we found first-degree adenoid hypertrophy in 10 (4.88%) children, second-degree in 112 (54.63%) children and third-degree in 83 (40.49%) children. In our series, there were no significant differences in the means of adenoid size or the Boleslavska scale between thermal seasons (*p* = 0.232, *p* = 0.565, respectively; Table 3). In contrast, when assessing the amount of mucus on the adenoids between thermal seasons, we observed deterioration in winter and an increase in the degree of mucus using our proposed MASNA scale. We found high statistical significance in seasonal dependence of mucus coverage of the adenoids (*p* = 0.000759). Middle ear effusion, confirmed by the results of tympanometry, also seems to be strongly associated with seasonality, as the number of incorrect tympanograms (type B or C) was significant higher in winter than in summer (*p* = 0.0000149) (Table 3).

## 4. Discussion

According to the medical experience of many ENT doctors, the number of upper respiratory infections in children decreases in summer. This has led to speculation that the size of the adenoids is also reduced in the summer.

Flexible endoscopy is the best method for assessing the adenoids in children, with a low rate of complications [8,9,10,19]. Unfortunately, it is not a routinely performed procedure in outpatients ENT clinics or hospitals because of its time-consuming nature, high cost, and the degree of experience required. However, it is possible to perform an assessment using a thin and flexible endoscope without anesthesia or premedication in more than 95% of children in age 2 to 12 years. It is essential to maintain contact with child patients, to allow play and to reward them. Parental collaboration and determination to obtain an objective diagnosis is also important, and pays off in correct diagnosis for oft-misdiagnosed patients and in a better possibility of using the most appropriate treatment.

Our study based on nasopharyngoscopy did not reveal any changes in adenoid size between the thermal seasons. There is no other study with which to compare results on seasonal change of adenoids in children based on flexible endoscopy; however, there are some works analyzing seasonal polysomnography (PSG) for the diagnosis of sleep-disordered breathing (SDB) and indirect estimation of changes of adenoid hypertrophy in children. Greenfeld et al. conducted a study in Israel based on polysomnography which showed that the seasonal difference in adenoid size was particularly significant in children younger than five years of age [12]. They stated that enlarged tonsils play an important role in sleep-disordered breathing (SDB) in children. They also suggested that their findings may indirectly support the role of the seasonal viral burden as a major determinant in season-dependent changes in SDB. Nakayama et al. undertook a similar study in Japan, which showed changes in the severity of SDB and snoring in different seasons [20]. Frimer et al. conducted a study in Israel in which they performed PSG and analyzed the prevalence of obstructive sleep apnea (OSA) in children in different seasons. However, they did not find any significant differences [2]. As the study of sleep, polysomnography deals with the air flow through the nasopharynx, but not its anatomical and functional structure.

The present study based on flexible nasopharyngoscopy revealed high volatility of adenoid mucus by season. Mucus provides a barrier against viruses and bacteria, while chronic inflammation may destroy this barrier and induce contamination [21]. Inflammatory nasal secretion has an impact on the severity of upper respiratory tract diseases because of the ability of microorganisms present in the secretion to multiply rapidly and produce a biofilm, leading to bacterial resistance against the host’s immune system. Biofilms most often form in humid and non-sterile environments. The multicellular bacterial structure surrounded by organic and nonorganic substances produced by the bacteria makes the biofilm cover porous surfaces, such as the tonsil [22,23]. The adenoid is a reservoir of microorganisms, including those with the ability to produce extracellular mucus [24,25]. Microorganisms from one or more species combine to form small colonies, which together constitute approximately 10% of the total volume of the biofilm [25]. The remainder includes extracellular polymeric substances (EPS), which keep the biofilm intact despite the adverse environmental conditions [23]. The glycocalyx is a particularly important substance in the EPS; it absorbs the necessary nutrients and promotes bacterial multiplication [24,25]. It is produced by bacteria such as *Pseudomonas* spp., *Moraxella* spp., or *Klebsiella* spp., which exist on the surface of the pharyngeal tonsil and can lead to the formation of non-sensitive bacterial colonies surrounded by thick mucus. Mucus-containing bacteria and viruses may contribute to the intensification of symptoms associated with adenoiditis [26]. Bellinghausen et al. state that pre-exposure of airway epithelial cells to pathological bacteria aggravates the production of proinflammatory cytokines in response to subsequent infections [27]. Clinical observations and clinical literature data confirm that the mechanical eradication of thick mucus by rinsing the nose with saline solution reduces the subjective and objective symptoms of adenoiditis [28].

In our opinion, the assumption of temperature dependence also undermines the theory of the influence of allergens and infections on the size of the pharyngeal tonsil, which corresponds with Greenfeld’s results [12]. Mucus on the pharyngeal tonsils seems to play a main role in the context of the exacerbation of the ailments related to the presence of the adenoids. This was also suggested by Nakayama et al. concerning the seasonal exacerbation of OSA in children [20]. Individuals allergic to certain environmental factors may present with seasonal allergies, which may impact adenoid size. However, most clinicians disagree on whether or not allergic rhinitis impacts adenoid hypertrophy [22,23,29,30,31,32,33]. Modrzyński et al. and Sadeghi-Shabesteri et al. described the relationship between adenoid hypertrophy and allergy related to dust mite hypersensitivity [22,29]. Dogru et al. suggested that only children hypersensitive to fungi may have seasonal adenoid hypertrophy, while those allergic to *Alternaria alternata* and dust mites do not [23]. In contrast, Karaca et al. did not find any correlation between adenoid size and skin prick test results in children aged 5 to 14 years old, but concluded that allergic hypersensitivity may play an important role in children with tonsillar hypertrophy [30]. Furthermore, Eren et al. and Ameli et al. found a negative correlation between allergies and adenoid size in their studies [31,32]. The study performed by Colavita et al. failed to demonstrate benefits of adenoidectomy in 80% of children with allergic rhinitis and hypertrophy, which seems to confirm the absence of or a negative correlation between allergies and adenoid size [33]. They concluded that only an endoscopic analysis of nasal secretions has significant predictive value for allergic rhinitis.

Our work based on performed tympanometry revealed seasonal tympanogram variability, and indicated seasonal change of otitis media with effusion (OME.) This is defined as an accumulation of fluid behind the intact tympanic membrane without signs and symptoms of acute ear infection in children, and seems to be strictly related to nasopharyngeal conditions such as chronic sinusitis and adenoid hypertrophy [34]. There are many postulated factors that may influenced OME, including age, allergies, breastfeeding, bottle feeding, presence of atopy or allergy, snoring, cough, more than five instances of tonsilitis in twelve months, presence of pets, attending a daycare center, passive smoking, number of siblings, family income, and seasonality [34,35,36]. For many years both otoscopy and tympanometry have been the gold standard for diagnosis of otitis media with effusion (OME). In 1981, Holm-Jensen showed great variations in repetitive tympanometry in four-year-old children when performed from winter to spring, which was connected with climate conditions [37]. In a continuation of this work based on tympanometry performed in 288 children, Mirko Tos found a significant increase in the frequency of otitis secretory and a significant deterioration in tympanometric condition in the winter [38]. For many years, OME was related with seasons [35]. In contrast to previously performed studies, Knopke in 2015 showed no significant seasonal difference between the winter and the summer period in intraoperative middle ear examination in connection with preoperative tympanograms [39].

This study has strongly demonstrated association between seasonality and OME as well as the co-occurrence of OME and increased amounts of nasopharyngeal mucus. The amount of mucus significantly changes between seasons. For describing mucus on the adenoid surface, we devised a scale called MASNA, which is useful for comparing the results of endoscopic examinations [40]. It may be speculated that it is difficult to compare the mucus coverage of adenoid in children, as some may blow their nose directly before the endoscopy and others may do not. Our experience shows that it is difficult for a child to eliminate deep-seated mucus from the nose during nasopharyngoscopy. This observation corresponds with parental opinion that it is impossible for their children to blow their nose despite the impression that the nose is blocked. One of the most useful methods for removing mucus is rinsing the nose with saline solution [41,42], which should thus be avoided prior to endoscopy.

## 5. Conclusions

The obtained results indicate that the thermal seasons do not influence the size of the pharyngeal tonsils. The increase and change in mucus coverage of the adenoids and deterioration of middle ear tympanometry in winter may be the cause of seasonal clinical deterioration in children instead of tonsillar hypertrophy. The proposed MASNA scale is useful for describing mucous coverage on the adenoids and for comparing endoscopy results. Flexible endoscopy is recommended and could be used more often for assessing the state of the adenoids in children. Further studies on the impact of the thermal seasons on adenoid size and mucus coverage, performed in different countries, are advisable.

## Figures and Tables

**Table 1:** Patient characteristics.

Characteristic		All Patients
*n*		205
Age at first visit (years)	mean ± SD	4.46 ± 1.56
	median (Q25–Q75)	4.00 (3.00–5.00)
Gender	female	99 (48.29%)
	male	106 (51.71%)
Sequence of examination	summer –> winter (S/W)	100 (48.78%)
	winter –> summer (W/S)	105 (51.22%)
Summer adenoid size	mean ± SD	62.17 ± 15.20
	median (Q25–Q75)	60.00 (55.00–75.00)
	grade I	9 (4.39%)
	grade II	118 (57.56%)
	grade III	78 (38.05%)
Winter adenoid size	mean ± SD	62.90 ± 16.27
	median (Q25–Q75)	60.00 (50.00–75.00)
	grade I	10 (4.88%)
	grade II	112 (54.63%)
	grade III	83 (40.49%)
Seasonal change in adenoid size	mean ± SD	0.73 ± 8.74
	median (Q25–Q75)	0.00 (−5.00–5.00)
	decrease	56 (27.32%)
	no change	76 (37.07%)
	increase	73 (35.61%)
Summer adenoid mucus coverage (MASNA scale)	0	47 (22.93%)
	1	82 (40.00%)
	2	53 (25.85%)
	3	23 (11.22%)
Winter adenoid mucus coverage (MASNA scale)	0	25 (12.20%)
	1	67 (32.68%)
	2	65 (31.71%)
	3	48 (23.41%)
Seasonal change in adenoid mucus coverage	decrease	51 (24.88%)
	no change	57 (27.80%)
	increase	97 (47.32%)
Summer tympanogram	AA	129 (62.93%)
	AB/BA	11 (5.37%)
	AC/CA	15 (7.32%)
	BB	23 (11.22%)
	BC/CB	11 (5.37%)
	CC	16 (7.80%)
	A	129 (62.93%)
	B	45 (21.95%)
	C	31 (15.12%)
Winter tympanogram	AA	86 (41.95%)
	AB/BA	6 (2.93%)
	AC/CA	17 (8.29%)
	BB	55 (26.83%)
	BC/CB	15 (7.32%)
	CC	26 (12.68%)
	A	86 (41.95%)
	B	76 (37.07%)
	C	43 (20.98%)

Data are presented as frequency (%) unless otherwise indicated.

**Table 2:** Relationship between sequence of examination and clinical and demographic variables.

Characteristic		The Sequence of Examination		*p* Value
		Summer –> Winter (S/W)	Winter –> Summer (W/S)	
*n*		100 (48.78%)	105 (51.22%)	
Age at first visit	mean ± SD	4.43 ± 1.54	4.49 ± 1.58	0.782
(years)	median (Q25–Q75)	4.00 (3.00–5.00)	4.00 (3.00–5.00)	
Gender	female	45 (45.00%)	54 (51.43%)	0.357
	male	55 (55.00%)	51 (48.57%)	
Summer adenoid size	mean ± SD	60.70 ± 15.75	63.57 ± 14.59	0.177
	median (Q25–Q75)	60.00 (50.00–72.50)	60.00 (60.00–75.00)	
	grade I	6 (6.00%)	3 (2.86%)	0.432
	grade II	59 (59.00%)	59 (56.19%)	
	grade III	35 (35.00%)	43 (40.95%)	
Winter adenoid size	mean ± SD	61.70 ± 16.18	64.05 ± 16.35	0.303
	median (Q25–Q75)	60.00 (50.00–75.00)	65.00 (55.00–80.00)	
	grade I	6 (6.00%)	4 (3.81%)	0.817
	grade II	54 (54.00%)	58 (55.24%)	
	grade III	40 (40.00%)	43 (40.95%)	
Seasonal change in adenoid size	mean ± SD	1.00 ± 9.24	0.48 ± 8.28	0.669
	median (Q25–Q75)	0.00 (−5.00–5.00)	0.00 (−5.00–5.00)	
	decrease	29 (29.00%)	27 (25.71%)	0.804
	no change	35 (35.00%)	41 (39.05%)	
	increase	36 (36.00%)	37 (35.24%)	
Summer adenoid mucus coverage (MASNA scale)	0	26 (26.00%)	21 (20.00%)	0.382
	1	42 (42.00%)	40 (38.10%)	
	2	24 (24.00%)	29 (27.62%)	
	3	8 (8.00%)	15 (14.29%)	
Winter adenoid mucus coverage (MASNA scale)	0	11 (11.00%)	14 (13.33%)	0.315
	1	38 (38.00%)	29 (27.62%)	
	2	32 (32.00%)	33 (31.43%)	
	3	19 (19.00%)	29 (27.62%)	
Seasonal change in adenoid mucus coverage	decrease	22 (22.00%)	29 (27.62%)	0.604
	no change	30 (30.00%)	27 (25.71%)	
	increase	48 (48.00%)	49 (46.67%)	
Summer tympanogram	AA	61 (61.00%)	68 (64.76%)	0.757
	AB/BA	7 (7.00%)	4 (3.81%)	
	AC/CA	8 (8.00%)	7 (6.67%)	
	BB	13 (13.00%)	10 (9.52%)	
	BC/CB	5 (5.00%)	6 (5.71%)	
	CC	6 (6.00%)	10 (9.52%)	
	A	61 (61.00%)	68 (64.76%)	0.576
	B	25 (25.00%)	20 (19.05%)	
	C	14 (14.00%)	17 (16.19%)	
Winter tympanogram	AA	49 (49.00%)	37 (35.24%)	0.254
	AB/BA	3 (3.00%)	3 (2.86%)	
	AC/CA	8 (8.00%)	9 (8.57%)	
	BB	21 (21.00%)	34 (32.38%)	
	BC/CB	5 (5.00%)	10 (9.52%)	
	CC	14 (14.00%)	12 (11.43%)	
	A	49 (49.00%)	37 (35.24%)	0.154
	B	29 (29.00%)	47 (44.76%)	
	C	22 (22.00%)	21 (20.00%)	

**Table 3:** Impact of the thermal season on adenoid size, adenoid mucus coverage and tympanograms.

Characteristic		Thermal Season		*p* Value
		Summer	Winter	
Adenoid size	mean ± SD	62.17 ± 15.20	62.90 ± 16.27	0.232
	median (Q25–Q75)	60.00 (55.00–75.00)	60.00 (50.00–75.00)	
	grade I	9 (4.39%)	10 (4.88%)	0.565
	grade II	118 (57.56%)	112 (54.63%)	
	grade III	78 (38.05%)	83 (40.49%)	
Adenoid mucus coverage (MASNA scale)	0	47 (22.93%)	25 (12.20%)	0.000759
	1	82 (40.00%)	67 (32.68%)	
	2	53 (25.85%)	65 (31.71%)	
	3	23 (11.22%)	48 (23.41%)	
Tympanogram	AA	129 (62.93%)	86 (41.95%)	0.0000149
	AB/BA	11 (5.37%)	6 (2.93%)	
	AC/CA	15 (7.32%)	17 (8.29%)	
	BB	23 (11.22%)	55 (26.83%)	
	BC/CB	11 (5.37%)	15 (7.32%)	
	CC	16 (7.80%)	26 (12.68%)	
	A	129 (62.93%)	86 (41.95%)	0.00000323
	B	45 (21.95%)	76 (37.07%)	
	C	31 (15.12%)	43 (20.98%)	
